# Improving Axial Resolution in Confocal Microscopy with New High Refractive Index Mounting Media

**DOI:** 10.1371/journal.pone.0121096

**Published:** 2015-03-30

**Authors:** Coralie Fouquet, Jean-François Gilles, Nicolas Heck, Marc Dos Santos, Richard Schwartzmann, Vidjeacoumary Cannaya, Marie-Pierre Morel, Robert Stephen Davidson, Alain Trembleau, Susanne Bolte

**Affiliations:** 1 Sorbonne Universités, UPMC Univ Paris 06, F-75005, Paris, France; 2 Institut de Biologie Paris-Seine, CNRS FR3631, F-75005, Paris, France; 3 CNRS, UMR8246, INSERM, U1130, Neuroscience Paris Seine, F-75005, Paris, France; 4 Citifluor Ltd, 18, Enfield Coisters, Fanshaw Street, London, N1 6LD, United Kingdom; University of Manchester, UNITED KINGDOM

## Abstract

Resolution, high signal intensity and elevated signal to noise ratio (SNR) are key issues for biologists who aim at studying the localisation of biological structures at the cellular and subcellular levels using confocal microscopy. The resolution required to separate sub-cellular biological structures is often near to the resolving power of the microscope. When optimally used, confocal microscopes may reach resolutions of 180 nm laterally and 500 nm axially, however, axial resolution in depth is often impaired by spherical aberration that may occur due to refractive index mismatches. Spherical aberration results in broadening of the point-spread function (PSF), a decrease in peak signal intensity when imaging in depth and a focal shift that leads to the distortion of the image along the z-axis and thus in a scaling error. In this study, we use the novel mounting medium CFM3 (Citifluor Ltd., UK) with a refractive index of 1.518 to minimize the effects of spherical aberration. This mounting medium is compatible with most common fluorochromes and fluorescent proteins. We compare its performance with established mounting media, harbouring refractive indices below 1.500, by estimating lateral and axial resolution with sub-resolution fluorescent beads. We show furthermore that the use of the high refractive index media renders the tissue transparent and improves considerably the axial resolution and imaging depth in immuno-labelled or fluorescent protein labelled fixed mouse brain tissue. We thus propose to use those novel high refractive index mounting media, whenever optimal axial resolution is required.

## Introduction

Imaging biological structures at the cellular and subcellular levels using confocal microscopy is often desired, but loss of resolution and decrease in fluorescence intensity in the depth of the sample are two important limiting factors. Imaging is particularly challenging in brain tissues, characterized by complex neural networks interconnected by synapses. To be in position of analysing the 3D organization of neural networks at the cellular level, neuroscientists usually work on relatively thick (e.g. 50 μm) vibratome sections and proper detection is required throughout the whole thickness of the section [1]. The resolution required for separating densely packed biological structures such as axons (the thinnest of them being 200 nm in diameter) and synaptic elements such as dendritic spines and synaptic boutons, which constitute neuronal connections is in the range of the resolving power of the confocal microscope. Theoretically, the lateral and axial resolutions that can be reached using confocal microscopes, when optimally used, are around 180 nm and 500 nm, respectively [2].

In optical microscopy, resolution is the shortest distance between two distinct points that can still be distinguished as distinct objects. The resolution of optical devices depends on two factors: the wavelength, and the numerical aperture of the objective lens, which is defined as the product of the sinus of the half-angle of the maximum cone of light that can enter or exit the lens and the refractive index (ri) of the medium. While the lateral resolution increases linearly with its numerical aperture, along the optical axis (z) the increase is quadratic.

In confocal microscopy, high numerical aperture oil-immersion lenses have become the standard for high-resolution imaging. They are designed and optimised to work with a glass coverslip that has to be introduced between the lens and the sample and a particular immersion medium that is oil. The refractive index of the glass-oil immersion system is close to 1.518 and offers a well-matched and optically homogeneous system with its focal point exclusively limited by diffraction. Optimally, the sample and mounting medium should have a matching refractive index.

However, this is rarely the case. Firstly, most biological samples contain water and may thus have a refractive index far from the oil-immersion objective. It has been stated though that alcohol or aldehyde fixation raises the refractive index of cells from approximately 1.350 to >1.500 [3]. Secondly, most standard sample preparation protocols for fixed samples like cultured cells, tissue sections of thick specimen as well as whole mount embryos include the embedding of the fixed biological material into mounting solutions mostly based on mixtures of glycerol or polyvinyl alcohol with water and various chemicals, i.e. antifading and preservation substances. According to information provided by the manufacturers, these media have refractive indices varying from ri = 1.450 to 1.490 and furthermore, some hardening media may even change their refractive index upon curing and reach at best a refractive index of 1.490.

It has been reported previously that the refractive index mismatch between the mounting medium and glass-oil immersion system results in a considerable loss of axial resolution in confocal microscopy due to spherical aberration [4, 5, 6]. Three phenomena can be observed: i) the point-spread function (PSF) broadens, ii) the peak signal intensity of the PSF decreases with penetration depth into the medium-sample system, iii) a focal shift leads to the distortion of the image along the z-axis and thus in a scaling error.

The use of adaptive optics has been proposed to counteract system and sample induced aberrations in confocal microscopy [7]. However, adaptive optics are not yet available on commercial confocal setups.

A nearby possibility to decrease spherical aberrations would be to use mounting media with a refractive index matching the glass-oil immersion system. This has been proposed by [8] who used 2,2’-Thiodiethanol, a water-soluble mounting medium with tuneable refractive index up to 1.520 for high-resolution optical microscopy. This study shows that the use of high refractive index media in combination with high numerical aperture objectives improves considerably axial resolution. New mounting media such as the glycerol-based CFM3 (Citifluor Ltd, UK) with refractive indices above 1.500 have been commercialized since then. Still they meet with little response, since many biologists use media with refractive indices below 1.500 for mounting fixed samples for immunocytochemistry. This might be a matter of habits or due to the reason that these mounting media are part of well-established immunocytochemistry protocols and that most of them have been experienced to work for a plethora of fluorochromes.

We aimed at testing those new mounting media with high refractive indices and therefore carried out a comparative study between conventional mounting media (ri <1.500) and high refractive index media (ri = or above 1.500). We measured the actual refractive indices, and studied their influence on lateral and axial resolution, determined with submicron fluorescent beads and in immuno-labelled mouse olfactory bulb. Furthermore, we used fixed brain sections from VGLUT1^Venus^ knock-in mice, in which synaptic vesicles are labelled with Venus [9] to determine maximal imaging depth in the different mounting media.

We demonstrated that while all mounting media tested performed well at the coverslip level, mounting media with a refractive index above 1.500 and matching exactly the glass-oil system resulted in a superior axial resolution, when looking at beads at a distance from the coverslip. We further observed that fixed mouse brain sections become immediately transparent when submerged into high refractive index mounting media, indicating that these mounting media match the refractive index of this fixed biological material. Interestingly, in contrast to conventional mounting media, the axial resolution measured within mouse brain slices incubated with high refractive index media remained constant throughout depth. When used for immunocytochemistry, these high refractive index mounting media greatly improved the axial resolution of immuno-labelled axons in the olfactory bulb of the mouse brain. Finally, these media displayed higher performance in imaging VGLUT1^venus^ labelled synaptic boutons throughout the working distance of the objective. We thus propose to use these high refractive index mounting media whenever optimal axial resolution is required.

## Results

We investigated the influence of mounting media with refractive indices below and above 1.500 on the lateral and axial resolution of the confocal microscope, using a high numerical aperture lens (63x, NA 1.4, oil immersion).

**Fig 1:**
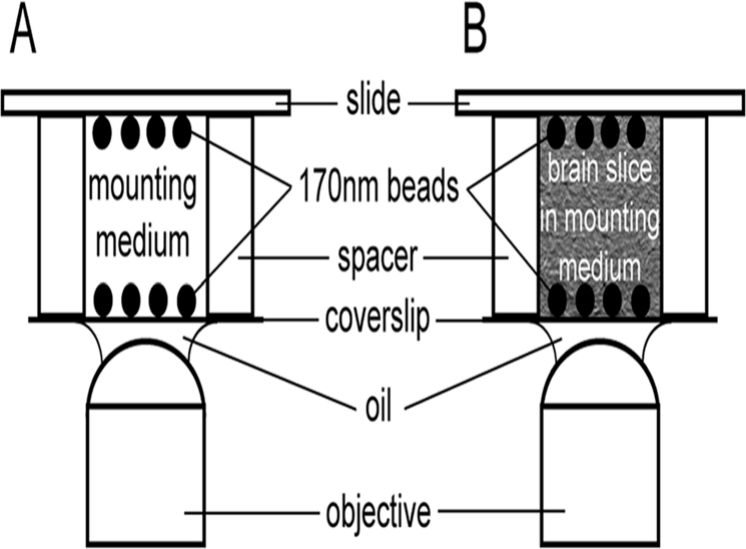
Experimental setup for evaluating axial resolution at different focusing depth with sub-resolution beads. (A, B) 170 nm green or red fluorescent beads have been immobilised on the coverslip and slide surface prior to mounting. One or two spacers of scotch tape (58 μm thick) have been introduced to obtain different depths. Coverslips have been assembled with mounting medium (A) or with brain slices incubated in mounting medium (B) and imaged with a 63x, 1.4 NA objective at 488 nm or 561 nm respectively.

To this end we determined the PSF, which is the impulse response of the focused optical system and which gives information about the lateral and axial resolution of the optical system. The PSF was measured by imaging 3D stacks of 170 nm fluorescent beads at different depths (Fig. 1) and characterised by its intensity profile. The lateral and axial resolutions were estimated from the full-width at half-maximum (FWHM) of the intensity profile of the PSF.

### 1. Lateral and axial resolution close to the coverslip

**Fig 2:**
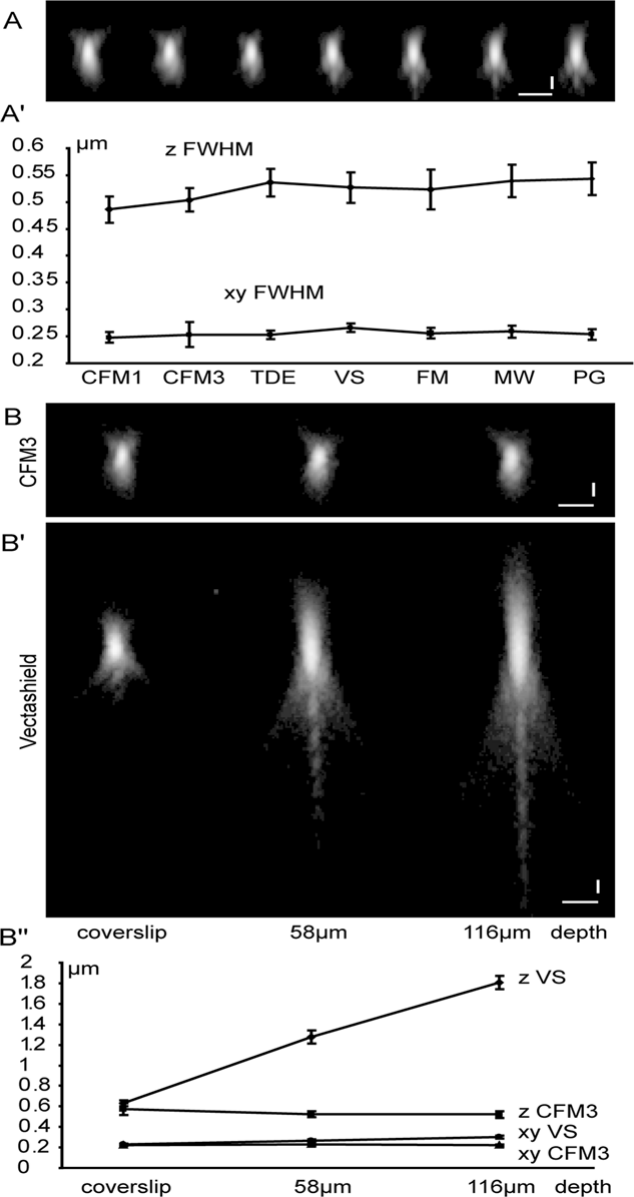
Lateral and axial resolution as a function of the mounting medium and focusing depth. 170 nm green beads have been mounted in CFM1, CFM3, 2’2-Thiodiethanol (TDE), Vectashield (VS), Fluoromount (FM), Mowiol (MW) or Prolong Gold (PG) mounting media as described in Fig. 1A, and measured directly at the coverslips (A, A’, B, B’) or in depth (B, B’, B”). Imaging was carried out with the 488 nm laser line collecting green emission. (A, A’) X, Z-projections of fluorescent beads are shown in A. Similar beads have been used to measure the lateral (dots) and axial (rhombus) resolution of the confocal microscope. Beads located at the coverslip show no significant difference in lateral (A’, mean value = 255 nm) and axial (A’, mean value = 523 nm) resolution through the different mounting media. Scale bar = 1 μm. (B, B’, B”) X, Z-projection of 170 nm beads mounted in CFM3 (B) or in Vectashield (B’) at the level of the coverslip, or at 58 μm or 116 μm depth. Axial resolution in high refractive index medium CFM3 (B”, triangles) remains constant with focussing depth, whereas axial resolution of beads increased with increasing focusing depth in Vectashield (B”, squares). Note that the lateral resolution remains unaffected in the two mounting media (B”, squares = CFM3 and circles = VS).

We first aimed at comparing seven commercially available mounting media with refractive indices ranging from 1.518 to 1.390, and determined the PSFs close to the coverslip. We imaged green fluorescent sub-resolution beads that were mounted directly on the coverslip, i. e. close to the objective lens (Fig. 2A, 2B). We determined a mean value of 255 nm for lateral and 523 nm for axial resolution with all tested mounting media observed. This set of data corroborated previous findings that close to the coverslip, a refractive index mismatch does not impair lateral and axial resolution in the media tested.

### 2. Lateral and axial resolution at a distance from the coverslip

We next analysed how the refractive index mismatch impairs resolution when looking at beads at a distance from the coverslip. We restricted our work on the comparison of the high refractive index mounting medium CFM3 (ri = 1.518) and the widely used Vectashield (ri = 1.454). We built a sandwich by mounting green fluorescent sub-resolution beads on slides and coverslips that were separated by one or two spacers of 58 μm 3. 1A). We determined the lateral and axial resolutions of beads mounted in CFM3 and Vectashield at the level of the coverslip and at 58 μm and 116 μm depth. Using CFM3, the lateral and axial resolution remained constant throughout depth (mean values x,y = 224 nm and z = 538 nm). This means that spherical aberration is minimized when using CFM3 medium, and our observation corroborated previous findings of Hell and co-workers for a perfectly matched system [4, 5, 6]. Lateral resolution in depth is not impaired in a mismatched system [4] and we indeed observed a lateral resolution of 220–240 nm for beads mounted in Vectashield up to a depth of 112 μm. However, axial resolution was doubled to > 1.2 μm (depth = 58 μm) and tripled to 1.8 μm (depth = 112 μm) when using Vectashield. Furthermore, we observed a considerable loss of peak intensity at a depth of 60 and 112 μm and had to adjust the laser power accordingly in order to exploit the full dynamic range of the image. These results confirm previous theoretical and experimental data for glycerol (ri = 1.480), showing that spherical aberrations occur because of refractive index mismatches in the depth of the sample [4, 5].

### 3. Influence of the penetration depth on the axial resolution in mouse brain vibratome sections

**Fig 3:**
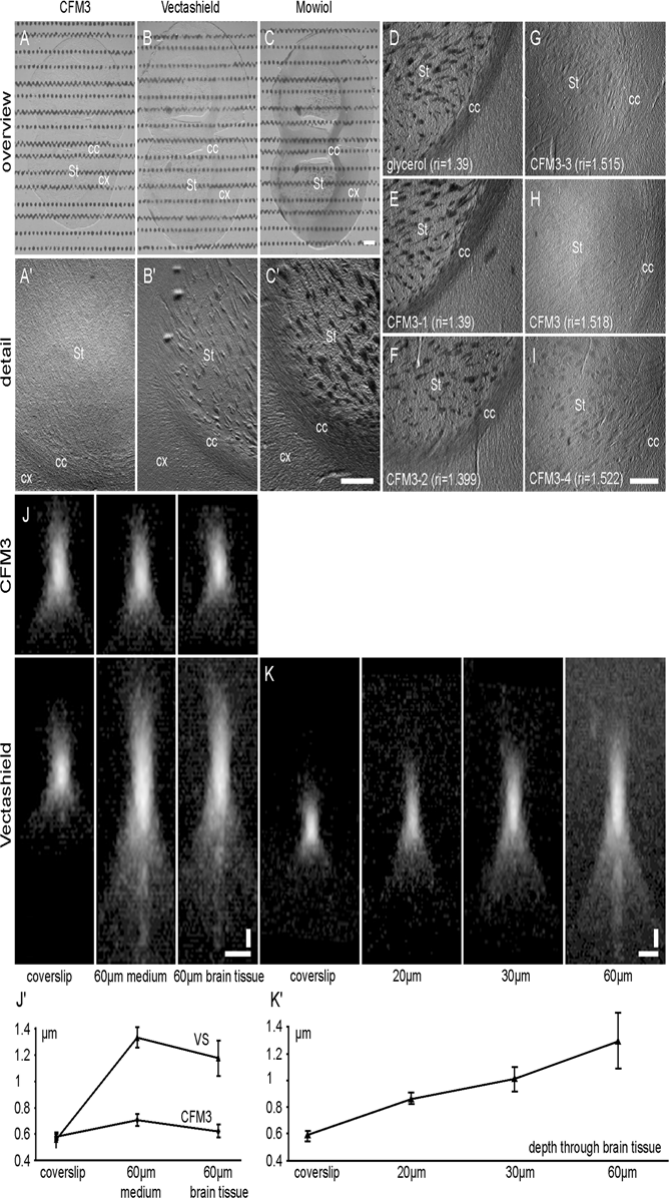
Axial resolution as a function of the mounting medium and focusing depth under brain sections. (A-I) 100 μm vibratome brain sections were mounted in different mounting media and bright field images were taken in relief-contrast mode at equal light levels with a 1.0 objective (Zeiss Axiozoomer). Overview image, taken with black and white pattern as background (A-C) and zoomed images (A’-C’, D-I) were carried out to visualise contrast changes in the striatum (S), corpus callosum (CC) and cortex (C). Sections were mounted in CFM3 (A, A’, H), ri = 1.518), Vectashield (B, B’, ri = 1.458), Mowiol (C, C’), glycerol (D, ri = 1.390), and CFM3–variants with tuned refractive indices, such as CFM3-1 (E, ri = 1.390), CFM3-2 (F, ri = 1.399), CFM3-3 (G, ri = 1.515) and CFM3-4 (I, ri = 1.522). Note that with fine-tuning of the refractive index to a value of 1.518, contrast of the brain section is very low. Scale bars = 500 μm. (J, J’) 170 nm red fluorescent beads were mounted in CFM3 (ri = 1.518) and Vectashield (VS), as described in Fig. 1B. Imaging was carried out with the 561 nm laser line collecting red emission. X, Z-projections (J) and axial resolution (J’) were assessed directly at the coverslip level (left), or at 60 μm depth, adjacent to brain sections (middle) and under the cortical region of the brain section (right). For beads mounted in CFM3 little variations in axial resolution were observed (J, upper panel, J’ circles, ri = 1.518). However, for beads mounted in Vectashield (J, lower panel, J’ triangles, ri = 1.454) a two-fold loss of resolution at 60 μm was observed. Scale bar = 1 μm. (K, K’) Red fluorescent beads were mounted in Vectashield (VS), as described in Fig. 1B. X, Z-projections (K) and axial resolution (K’) was measured directly at the coverslip level (left) and below the cortical region of brain sections, at 20 μm, 30 μm and 60 μm depth. Note that a considerable loss of axial resolution is already observed at 20 μm depth. Scale bar = 1 μm.

We next aimed at performing a comparative study of different mounting media on a biological tissue. We carried out 100 μm thick vibratome sections of fixed mouse brains and mounted them in CFM3, Vectashield or the hardening mounting medium Mowiol. Images were taken with an Axiozoomer V16 equipped with a 1x objective in relief contrast mode (Fig. 3A-3C). This technique is based on Hoffman modulation contrast, which converts phase gradients into variations in light intensity [10]. We observed a reduction of phase contrast right after immerging brain sections in CFM3, which rendered the mouse brain almost completely transparent (Fig. 3A, 3A’). Instead, brain sections mounted in Vectashield or Mowiol remained quite contrasted (Fig. 3B, 3B’, 3C, 3C’). These differences appeared particularly striking in brain areas in which myelinated axon bundles are present, i.e. the corpus callosum, and the striatum (Fig. 3A-3I).

We aimed to be sure that the transparency of mouse brain sections when mounted into CFM3 is due to refractive index matching of the fixed brain tissue with the medium and not to mounting medium components. We therefore used CFM3-variants (thereafter labelled CFM3-1 to CFM3-4) with refractive indices tuned to 1.390, 1.399, 1.515 and 1.522 by varying the amounts of glycerol and water to the CFM3 solution. Furthermore, we used a glycerol/water solution (ri = 1.390) to mount brain sections. We observed that glycerol (ri = 1.390), CFM3-1 (ri = 1.390) and CFM3-2 (ri = 1.399) resulted in comparable images of the brain sections (Fig. 3D-3F). Striatal fibres and corpus callosum were very contrasted. Fine-tuning of the refractive index of CFM3 showed that with the original CFM3 (ri = 1.518) the contrast is completely lost, whereas CFM3-variants with ri-values differing only slightly by 0,003 ± 0,001 from CFM3 increase notably the contrast (Fig. 3G, 3I). This set of data clearly demonstrates that the transparency observed with CFM3 is due to refractive index match of the fixed brain section with the medium.

Mouse brain vibratome sections of 60 μm thickness were then introduced between slides and coverslips prepared with orange fluorescent beads on both sample directed sides (Fig. 1B). PSF-measurements were subsequently carried out in three positions, at the level of the coverslip, in a depth of 60 μm adjacent to the brain slice, and in a depth of 60 μm beneath the cortical region of the brain (Fig. 3J, 3J’). In the refractive index matched systems with CFM3 mounting medium, the axial resolution remained constant (mean value 635 nm) between the coverslip, and in depth adjacent and beneath the brain section. When using Vectashield as a mounting medium, refractive index mismatch between the oil-glass interface and between the mounting medium-sample interfaces occurs. This mismatch leads to a two-fold reduction in axial resolution at 60 μm sample thickness adjacently and beneath the brain section. These results further demonstrate that the refractive index of fixed brain sections and CFM3 matches perfectly, and that under these conditions spherical aberrations are absent.

When performing immuno-labelling of mouse brain sections, antibody labelling penetrates rarely deeper than 30 μm into the sample. We thus wanted to know if our findings were consistent in sample depths relevant to immuno-labelling. We therefore investigated the axial resolution at sample thicknesses of 20, 30 and 60 μm using Vectashield as a mounting medium. In this medium, we observed a resolution loss of 30% already in a depth of 20 μm (Fig. 3K, 3K’).

### 4. Improvement of axial resolution in immunofluorescence

**Fig 4:**
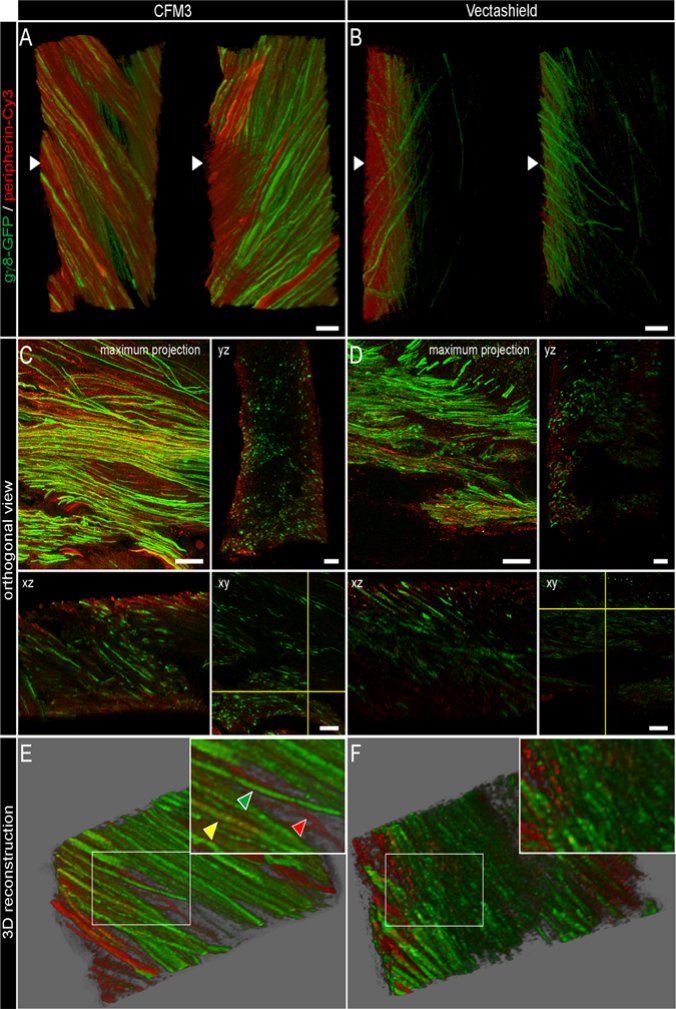
Improvement of axial resolution in fixed mouse olfactory bulbs. Fifty-micron thick vibratome sections of olfactory bulb from adult transgenic mice (Gg8-TTA x Tet0M72iresGFP) were processed for double immunofluorescence detection of GFP (green) and Peripherin (red) and mounted subsequently with CFM3 (A, C, E) or Vectashield (B, D, F). Confocal datasets were deconvolved. (A, B) 3D-reconstruction side view of characteristic z-stacks of brain sections mounted in CFM3 (A) or Vectashield (B) with the coverslip position on the left (arrowhead). (C, D) Orthogonal slices of z-stacks taken from sections mounted in CFM3 (C) or Vectashield (D) to demonstrate axial resolution and signal intensity throughout a single section (x, y-view, x, z-view and y, z-view) and in a maximum projection of 20 images (maximum projection). (E, F) 3D maximum projection of 8 confocal sections through olfactory bulb slices mounted in CFM3 (E) or Vectashield (F), with the insert showing a zoomed view. Note that in brain sections mounted in CFM3, 3 categories of axons can be easily distinguished: GFP+/peripherin- axons (green arrowhead), GFP-/peripherin+ axons (red arrowhead), and GFP+/peripherin+ axons (yellow arrowhead). The images obtained using Vectashield do not allow such a clear distinction between these differently labelled subpopulations of axons. Scale bars = 10 μm.

We aimed at assessing the ability of our new protocol, involving CFM3 as a mounting medium, to provide higher axial resolution than conventional media like Vectashield in immunofluorescence labelling. We therefore decided to use the olfactory sensory axons of the mouse as a test system. These axons, originating from the olfactory epithelium lying in the nasal cavity, project to the brain at the level of the olfactory bulb. On their way towards their targets in the glomerular layer, these axons rearrange in the most peripheral layer of the bulb, called the olfactory nerve layer [11]. In this layer, olfactory axons are highly numerous and packed at a very high density, making them extremely difficult to resolve by light microscopy. To label olfactory axons, we used transgenic Gγ8-TTA x TetO-M72iresGFP mice [12, 13] expressing GFP in all immature olfactory sensory neurons, through the use of a Gγ8 promoter. To test whether our protocol would be suitable for co-localization studies, immunofluorescence against GFP and peripherin was performed. Peripherin is an intermediate filament known to be expressed in olfactory axons, and previously described to be expressed with a distinct spatiotemporal pattern in these axons [14, 15]. We document in Fig. 4 the difference, in terms of quality of imaging (in depth sensitivity and axial resolution) between Vectashield and CFM3 used as mounting media. All images were taken from the olfactory nerve layer of the olfactory bulb that contains essentially fascicles of olfactory axons en route towards their targets. As seen on Fig. 4A,B, CFM3-mounted sections allow the detection of the labels throughout the thickness of the section for both markers, whereas Vectashield-mounted sections allows the visualization of the labels only in superficial zones of the sections close to the coverslip. Immuno-labelling was carried out in a floating manner and labelling should thus be observed throughout the section even in Vectashield mounted preparations. Correct labelling of the opposite site was verified by turning the section (unpublished data). As shown in orthogonal views (Fig. 4C, 4D), while Vectashield-mounted sections display a poor resolution of the labelling, as observations are made deeper in the section, this is not the case with CFM3-mounted sections, which clearly display a very high resolution of labelling, with excellent and homogeneous sensitivity, throughout the thickness of the sections. Indeed, only CFM3 allows a satisfactory visualization of the thin (200 nm diameter) densely packed axons in both longitudinal and orthogonal views (Fig. 4A, 4C). A maximum intensity projection of 8 sections illustrates the high quality of images obtained from CFM3-mounted sections in which individual axons can be delineated (Fig. 4E). In contrast, Vectashield-mounted sections retrieved satisfactory resolution only in the few first μm of tissue depth (Fig. 4F). Since Peripherin localizes in the olfactory nerve layer with a distinct spatiotemporal pattern, with differential localization along these axons in the outer and inner olfactory nerve layer [15], and since in the transgenic mice GFP is expressed only in a subset of olfactory axons [12, 13], we expected to observe individual axon profiles expressing either marker, and some both. Using CFM3, regardless of the small diameter of olfactory axons, close to the limit of resolution of light microscopy, we can clearly resolve 3 categories of axons: GFP+/peripherin- (green) axons, GFP-/peripherin+ (red) axons, and GFP+/peripherin+ (yellow) axons (Fig. 4E).

Our results clearly show that only CFM3-mounted sections allow excellent in depth imaging (Fig. 4A), and optimal and constant axial resolution throughout the entire thickness of the 50 μm sections (Fig. 4C, 4E). In addition, CFM3 demonstrated its full suitability for co-localization purposes, even at this 200 nm scale, throughout the entire thickness of the sections (Fig. 4E, inset).

### 5. Improvement of axial resolution when imaging a fluorescent protein

**Fig 5:**
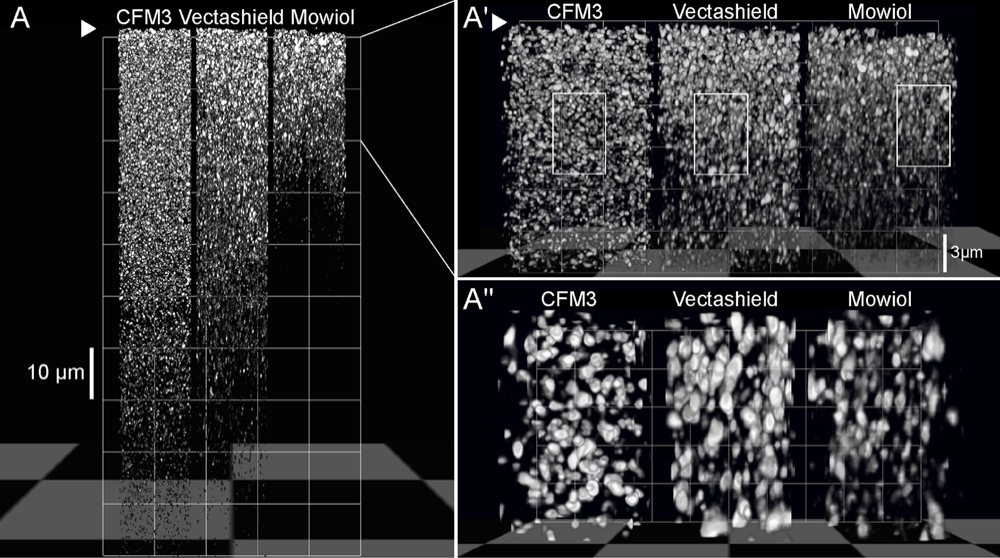
Depth penetration and resolution improvement in VGLUT1-Venus mouse brain slices. 100 μm vibratome sections of brain of adult transgenic mice (VGLUT1^Venus^) were mounted in CFM3, Vectashield or Mowiol and imaged using the 514 nm laser line. Imaging was carried out using an optimal z-section. 3D-reconstructions of the entire z-stack (A) with the coverslip position at the top (arrowhead) and the first 20 μm (A’) of VGLUT1^Venus^-labelled synaptic boutons were carried out to demonstrate depth penetration in the different media. (A”) is a magnified view of the insert in (B) showing synaptic boutons in a depth of 5–15 μm. Note that the boutons mounted in Vectashield appear elongated in comparison to CFM3 mounted sections. Scalebars are 10 and 3 μm.

In order to estimate the loss of intensity at different depths of the tissue, sections with homogeneous distribution of fluorescence are required. We thus used cortical sections from VGLUT1^venus^ knock-in mice, in which the gene coding synaptic marker VGLUT1 is fused with gene coding fluorescent protein Venus, so all presynaptic boutons expressing VGLUT1 are fluorescent [9]. Since the density of VGLUT1-positive synapses within a layer of the neocortex is homogenous [16], we could estimate the signal intensity profile through section depth. We imaged z-stacks of 100 μm sections of fixed brain, mounted in CFM3, Vectashield and Mowiol (Fig. 5). VGLUT1 positive presynaptic boutons are blobs of 300 to 900 nm diameter and are evenly distributed through the depth of the section. Subsequent 3D-reconstructions show that depth penetration is best in CFM3 mounted sections, where well resolved synaptic boutons are observed throughout the section until a depth of 80 μm (Fig. 5A). In Vectashield mounted sections synaptic boutons are observed until a depth of 50 μm, and in Mowiol mounted sections depth penetration does not exceed 30 μm. We then monitored synaptic bouton shape throughout the first 30 μm of the sections (Fig. 5A’) where we observe that in CFM3-mounted sections the shape of the boutons is well preserved along the z-axis and that the boutons are well resolved. In Vectashield and Mowiol mounted sections boutons appear elongated already in a depth of 5–15 μm (Fig. 5A’, 5A”) and they appear to be less well resolved.

## Discussion

Using CFM3, a commercially available mounting medium with a refractive index of 1.518, which matches exactly the refractive index of the glass-oil immersion system, we report a considerable improvement of axial resolution when imaging fixed samples in depth with confocal microscopy.

It has been shown that the axial PSF broadens and that its intensity profile decreases drastically with penetration depth when a mismatch between the glass-oil immersion system and the mounting medium and sample occurs, whereas lateral resolution remains unaffected [4, 5, 6]. In those studies, comparisons between a perfectly matched systems (immersion oil) and mismatched systems with air, glycerol (ri = 1.480), or water (ri = 1.330) have been carried out. It has been shown that aberrations can be avoided and fluorescence collection optimised only when refractive indices are matched correctly.

Staudt and co-workers have proposed the use of 2,2’-Thiodiethanol (TDE), a water-miscible and tunable mounting medium to obtain an optimally matched system to mount cells [8]. Although excellent results can be achieved with TDE, it should be noted that its preparation requires pH adjustment and its hygroscopic property renders it instable unless stored in controlled water-free environment [8]. On the other hand, the ready-to-use, stable CFM-3 ensures reproducible results. One important property a mounting medium should fullfill is a full compatibility with all fluorochromes. TDE is based on sulfides, while CFM is based on sulfoxides. It is known that unlike sulphides, sulfoxides show little tendency to quench excited singlet (and hence fluorescence) and triplet states because of their higher oxidation potential [17]. Thus, while the use of TDE is restricted [8], CFM-3 is compatible with most fluorochromes.

We compared the impulse response of the confocal system by measuring the PSF with fluorescent sub-resolution beads mounted in CFM3, with a high refractive index equal to glass (ri = 1.518) and other commercially available mounting media with refractive indices below 1.500, such as Vectashield (ri = 1.458). We observed indeed, that in the perfectly matched system (CFM3) the axial resolution remains invariant over the complete working distance of the objective. However, in our experimental situation, we observe a decrease in resolution by a factor 2 in a depth of 58 μm for the mismatched system using Vectashield and a 1.4 NA objective. It has been assumed, based on theoretical results, that the axial resolution decreases by a factor 1.2 per 10 μm when using a 1.3 NA objective and supposing a refractive index of 1.470 [4]. This would lead to 1.8 μm axial resolution at 60 μm depth and thus to an overall threefold decrease in axial resolution when using 170 nm fluorescent beads, which is in a comparable range as our experimental results.

We observed that fixed mouse brain sections become immediately transparent when plunged into CFM3-mounting medium, having a refractive index of 1.518. However, when using CFM3 variants with a refractive index varying by 3 or 4 in the last decimal point, brain sections remained slightly opaque and image contrast was preserved, when using Hoffmann contrast mode. Refractive index tuning was carried out by adjusting the concentrations of water and glycerol in order to exclude that other components of the mounting medium have an effect on transparency. One of the mechanisms responsible for the observed loss of contrast might thus be the matching of the refractive indices between the medium, which replaces the water, and cellular components such as macromolecular complexes as it has been described for some optical clearing agents [18]. The refractive index of fresh mouse brain has been studied by various methods and lies between 1.355 and 1.400 [19, 20, 21]. To our knowledge, no refractive index data is available for the fixed mouse brain. In flow cytometry, an increase in refractive index upon fixation is used to improve light scattering measurements helping to discriminate between cell types [22]. Furthermore, it has been shown by near field penetrating optical microscopy, that alcohol or aldehyde fixation raises the refractive index of cells from 1.350 to over 1.520 [3]. In addition, optical clearing protocols relying on refractive index matching for the mouse brain are based on high refractive index substances such as urea or fructose [23, 24]. Finally, we observed that the axial resolution measured with beads in CFM3-embedded mouse brain sections remained constant until 60 μm imaging depth. However, a considerable loss of resolution in the mismatched system (Vectashield) occurred already in a depth of 20 μm. Spatial invariance of the PSF in the matched system hints to the absence of spherical aberration and corroborates our suggestion that CFM3 (ri = 1.518) is very close to the refractive index of the macromolecular components of the fixed mouse brain.

We then compared the performance of CFM3 and Vectashield media on 50 μm thick, fixed, immuno-labelled mouse brain vibratome sections. We demonstrate that we can image densely packed, thin axons with a diameter of 200 nm with excellent resolution in all three dimensions when using CFM3-medium. This was achievable throughout the entire thickness of the 50 μm thick vibratome sections used here, making it now possible to assess molecular and organizational features of these axons that were hardly accessible without the use of electron microscopy at the light microscope level before [11]. In Vectashield mounted samples, a sufficient and neat immuno-fluorescence signal of the brain sections can be observed until a depth of 25 μm, however, further depth penetration seems to be impaired due to spherical aberration.

We performed confocal microscopy on thick brain sections (100 μm) densely labelled with VGLUT1^venus^. VGLUT1-boutons were properly imaged up to a depth of 80 μm. However, we observed a slight reduction in the intensity of the fluorescence signal along the z-axis. This may be due to two phenomena: Firstly, brain is a strongly scattering tissue and furthermore the labelling density of VGLUT1 is very high. This may lead to light scattering of the fluorescent signal in the volume and thus to less photons that reach the detector. Secondly, Venus fluorescent protein seems to be slightly more sensitive to photo bleaching in CFM3 mounting medium compared to Vectashield or Mowiol mounting medium (data not shown). Increased photo bleaching of fluorescent proteins has been already observed for other high refractive index mounting media [8] as well as for Prolong Gold (Table 1). However, although photo-bleaching was less pronounced in Vectashield or Mowiol mounted sections, depth penetration was already impaired at 50 and 30 μm, respectively, and axial resolution of the synaptic boutons was only appropriate in the first 20 μm and decreased rapidly afterwards.

**Table 1:** Comparison of mounting media.

****Mounting medium****	****Manufacturer****	****Refractive index****	****pH****	****Base compound****	****Fluorophore compatibility**** *
2,2'-Thiodiethanol	Sigma-Aldrich, France	1.518	6.5	2,2'-Thiodiethanol	most organic dyes and fluorochromes, RFP, however causes strong GFP quenching (8)
CFM-1	Citifluor Ltd., UK	1.515	7.5	glycerol-PBS-based	nd
CFM-3	Citifluor Ltd., UK	1.518	6.5	glycerol-based	DAPI, Hoechst, Alexa and Cyanine dyes, Venus, GFP^#^, Tomato, mCherry
Vectashield H-1000	Vector Laboratories Ltd., UK	1.454	nd	glycerol-based	fluorescein, rhodamine, Texas Red, AMCA, DyLight fluorescent dyes and other fluorescent reagents such as Cy3, Cy5, Alexa Fluor 488, and Alexa Fluor 594. GFP, RFP, YFP
Prolong Gold	ProLong Gold	1.390–1.460 depending on curing time	nd	glycerol-based	most organic dyes, however, fluorescent proteins less well preserved
Fluoromount-G	Southern Biotechnology	1.400	7.4	acrylate-PBS	most organic dyes
Mowiol Tris MWL 4–88	Citifluor Ltd., UK	1.410–1.490	9.5	polyvinyl alcohol	most organic dyes

*fluorochrome compatibility was experimentally evaluated only for CFM3, data for other mounting media were taken out of the manufacturers datasheets.

^#^ GFP shows enhanced photobleaching in CFM3

## Conclusions/Perspectives

CFM3-mounting medium provides an important benefit to confocal imaging of fixed samples with a high numerical aperture lens because it matches exactly the refractive index of the glass-oil system. Here, we show that CFM3 matches the refractive index of fixed mouse brain sections, which become completely transparent when mounted into this medium. Refractive index matching is particular important to avoid spherical aberration and thus improve axial resolution and signal strength of the confocal system in depth. Furthermore, spatial invariance of the PSF in depth is also crucial to improve deconvolution, since axial shift invariance is assumed in most algorithms [25].

CFM3 is compatible with various organic fluorochromes and the fluorescent protein Venus. Preliminary results show that a variety of other fluorescent proteins are also compatible with this mounting medium, even though photo bleaching may occur especially with GFP (Table 1). We are currently investigating new variants of CFM-media and their compatibility with various fluorescent proteins.

## Materials and Methods

### Mounting media

We compared seven mounting media with refractive indices below and above ri = 1.500, to test different refractive index mismatch conditions. Refractive indices for non-hardening mounting media were verified at 21°C using a refractometer (Pal-RI, Cat.-number: 3850, ATAGO, Japan) (Table 1).

Mounting solutions below ri = 1.500 were Vectashield (Vector laboratories) with a measured refractive index of ri = 1.454, Fluoromount-G (Southern Biotech Assoc, ri = 1.389), Prolong Gold (Life Technologies), Mowiol (Tris-MWL 4–88, Citifluor) and a glycerol/water-mixture (ri = 1.390). Following manufacturer’s recommendation, curing of the Prolong was carried out at room temperature for 48h so its refractive index increases and stabilizes around ri = 1.440. The refractive index of Tris-MWL 4–88, a mixture of Mowiol, Tris and glycerol/water seems to range between 1.410 and 1.490. The refractive index of many sulfoxides such as methyl phenyl sulfoxide is quite high (ri = 1.5775). The use of these and related compounds has been explored in an attempt to produce a widely applicable mounting medium (R S Davidson UK Patent 2006 GB2419427). Two of those mounting media above ri = 1.500 were glycerol-based CFM1 (Citifluor, ri = 1.515) and CFM3 (Citifluor, ri = 1.518) containing propyl-gallate as an antifadent. Furthermore, 2,2’-Thiodiethanol (Sigma) has been prepared as described in [8] to reach a refractive index of ri = 1.518. CFM3-variants with refractive indices tuned from 1.390–1.522 were produced by varying the amount of water and glycerol.

### Preparation of Sub-resolution fluorescent beads and Experimental setup for evaluating the axial resolution in depth

Fluorescent beads (PS-Speck, Lifetechnologies), of a diameter of 170 nm, and loaded with yellow-green and orange fluorescent dye, were diluted in water (1/800 v/v). Drops of the water-diluted sample were put on the surface of the coverslip (Menzel Glaeser #1.5, Agar scientific) or slide and air-dried. Beads were then mounted with a drop of mounting medium. Two experimental setups for evaluating axial resolution in depth were designed. First, fluorescent beads were mounted on coverslips and slides that were separated by one or two layers of adhesive tape (Scotch, 3M) with a nominal thickness of 58 μm and the volume filled with a drop of the respective mounting medium (Fig. 1A). Secondly, orange fluorescent beads were mounted on coverslips and slides, and mouse brain slices of different thickness (15, 30, 60 μm) incubated in CFM3 or Vectashield were inserted in between (Fig. 1B).

### Confocal laser scanning microscopy

8-bit Images were collected using a Leica 63x oil immersion objective (HCX Plan APO CS, NA 1.4, working distance 0.14 mm) with an inverted Leica laser-scanning confocal microscope TCS SP5 II (Leica Microsystems, Heidelberg, Germany) equipped with a GaAsP hybrid detection system at a sampling rate of 60 nm in x,y and 200 nm in z-direction. Fluorochromes were detected using laser lines 488 nm and 514 nm. Imaging was performed in a temperature-controlled room at 21°C.

### Image acquisition of beads

Bead images were obtained as in [26] with the following modifications. GaAsP gain was set to 16% and the laser power adjusted so that the signal occupied the full dynamic range of the detector, but saturated voxels were carefully avoided. Beads were imaged starting and finishing the stack at least 5μm below and above the bead centre. Beads were visually checked and improper stacks were discarded before determining the microscope PSF. At least eight imaged beads were registered and averaged in order to increase the SNR for deconvolution. Measured PSFs presented in Figs. 2 and 3 show representative beads displayed on the same logarithmic scale so that the low intensity detail characteristics of diffraction patterns was enhanced.

### Data analysis

Determination of the lateral and axial resolution was carried out with the ImageJ-based MetroloJ plugin for ImageJ [27, 28]. The plugin generates maximum intensity projections of the stack along the x, y and z-axis resulting in 1D intensity profiles. The (x, y) coordinates of the **m**aximum **i**ntensity **p**ixel (mip) are then collected. A x,z cross-section is generated along a line passing through the previously determined 2D mip. From this image, the z coordinate of the mip is defined. The z slice is set to the z mip coordinate. The x profile and y profile are collected along the line passing through the mip. The z profile is collected on the x,z view, along the line passing through the mip. All three profiles are fitted to a Gaussian curve, using ImageJ’s built-in curve fitting function. The full width at half maximum (FWHM) of the gauss curve is calculated for each profile, based on the parameters retrieved from the fitting.

### Deconvolution and image treatment

Confocal images of biological data of Figs. 4 and 5 were deconvolved with the Huygens 3.7 software (Scientific Volume Imaging, Hilversum, Netherlands) using a measured PSF (see above) and the Classical Maximum Likelihood Estimation algorithm with 100 iterations. Signal-to-noise ratios (SNR) lower than 20 or up to 50 are recommended by the manufacturer for noisy confocal images or low noise wide-field images. Since we used a confocal microscope with a low noise detector, we tested signal to noise ratios (SNR) of 15, 17, 19, 21 and 25 and visually inspected the results. A signal to noise ratio of 19 gave best results after visual inspection of the raw versus the deconvolved images. In images of Fig. 4 brightness and contrast were adjusted equally for all images after deconvolution and before 3D reconstruction, orthogonal slicing and volume rendering using ImageJ [27].

### Preparation of mouse brain sections

Adult Gg8-TTA x TetO-M72iresGFP mice [10, 11] and VGLUT1^venus^ mice [9] were anesthetized with sodium pentobarbital (50 mg/kg i.p.) and fixed by intracardiac perfusion of 4% paraformaldehyde in 0.12 M phosphate-buffer saline (PBS), pH 7.4. The brain was removed, then postfixed for 3 h in the same fixative and kept in PBS at 4°C until vibratome sectioning. The olfactory bulbs of Gg8-TTA x TetO-M72iresGFP mice were cut in the frontal plane, and processed for immunolabeling. Frontal sections of VGLUT1^venus^ mice were directly observed since these mice express synaptic marker VGLUT1 fused to the fluorescent protein Venus. Sections of various thicknesses were collected serially in PBS. For their mounting, sections were incubated for 5 minutes in the respective mounting medium and mounted directly on the coverslip. Coverslips were then mounted on slides and exceeding mounting medium was squeezed out by tapping onto the coverslip. Confocal x,z-scans were performed to make sure that the brain sections were positioned directly adjacent to the coverslip.

Animal care was conducted in accordance with standard ethical guidelines (NIH publication no. 85–23, revised 1985 and European Committee Guidelines on the Care and Use of Laboratory Animals 86/609/EEC), and the experiments were approved by the local ethic committee “Comité d’Ethique en Expérimentation Animale Charles Darwin C2EA–05” (Ministère de l’Enseignement Supérieur et de la Recherche, Paris, France).

### Immunocytochemistry

Sections were blocked for 1 h in PBS containing 0.25% Triton-X, 0.2% gelatine, 0.1% sodium azide and lysine (0.1 M) before applying primary antibody in PBS containing 0.25% Triton-X100, 0.2% gelatin, 0.1% sodium azide overnight on floating sections. Primary antibodies were chicken anti GFP (1/500, Aveslab) and rabbit anti-Peripherin (1/100, Charles Greer’s gift).

Sections were washed in PBS containing 0.25% Triton-X, and then incubated for 2 h with species-specific secondary antibodies, donkey anti-chicken linked to Alexa-488 (1/400; Invitrogen) and donkey anti-rabbit linked to Cy3 (1/200, Jackson) in PBS containing 0.25% Triton-X100, 0.2% gelatine, 0.1% sodium-azide. The sections were thereafter washed several times in PBS and mounted in CFM3 or Vectashield.
